# Impact of Photobiomodulation on the Quality of Life of Men and Women With Androgenetic Alopecia

**DOI:** 10.7759/cureus.66023

**Published:** 2024-08-02

**Authors:** Leonardo de Medeiros Quirino, Tania Maria da Silva Mendonça, Carlos Henrique Martins da Silva, Helena Borges Martins da Silva Paro

**Affiliations:** 1 Medicine, Federal University of Uberlandia, Uberlandia, BRA

**Keywords:** female pattern hair loss, male pattern hair loss, hair loss treatment, quality of life, low-level light therapy, photobiomodulation, androgenetic alopecia, alopecia

## Abstract

Objectives: Androgenetic alopecia (AGA) is the most common cause of hair loss in men and women, and it can affect the psychological and social activities of individuals, thus reducing their quality of life. Photobiomodulation (PBM) is a recent adjuvant treatment for this condition with promising results for hair regrowth. We aimed to assess the health-related quality of life of men and women with AGA before and after PBM sessions.

Methods: This is a single-center prospective observational study conducted with 42 men and 43 women with AGA. All participants answered a sociodemographic questionnaire in an interview and individually answered the Brazilian version of Skindex-29 (self-application). After 24 PBM sessions, two 20-minute sessions per week, with 48 to 72 hours of interval between sessions, participants answered the Skindex-29 again.

Results: Women had a large reduction in Skindex-29 total score after PBM (p<0.01; d=0.82) and lower scores in the emotions (p<0.01; d=0.89), psychosocial functioning (p<0.01; d=0.60), and symptoms domains (p=0.03; d=0.38). Men presented a moderate reduction in Skindex-29 total score after PBM (p<0.01; d=0.68), largely lower scores in the emotions domain (p<0.01; d=0.82) and a small reduction in the psychosocial functioning domain (p<0.01; d=0.47).

Conclusions: The use of PBM in AGA is associated with improving the quality of life of men and women. This enhancement was higher regarding emotions, the major domain affected in the AGA population. Women had larger impacts on all domains of Skindex-29 after the use of PBM.

## Introduction

Androgenetic alopecia (AGA) is the most common cause of hair loss in men and women. As hair plays an important role in body image, AGA can affect the psychological and social activities of individuals, thus reducing their health-related quality of life (HRQoL) [1,2]. Patients who suffer from this condition feel anguished about their lives and the way others see them [3]. Although AGA is a mild dermatological disorder, psychologists and dermatologists have observed that even clinically imperceptible hair loss can impair the HRQoL of affected patients due to loss of self-image and decreased self-esteem[3,4]. In women with AGA, 88% showed impaired effects on their routine life; 75% demonstrated low self-esteem and half suffered social problems [5], while about 25% of men with AGA were unsatisfied with their self-image and 62% reported emotional distress [6].

New adjuvant therapies for AGA have emerged, such as photobiomodulation (PBM). It presented promising results for hair growth and received FDA approval in 2007 and from the Brazilian regulatory agency ANVISA in 2016. It has been rapidly gaining popularity due to its ease of use and absence of side effects [7,8].

It is important to study the HRQoL in individuals with AGA considering the chronicity of the condition and the possible side effects in the first choice therapeutic options [9].

Although the scientific literature has shown improvement in the HRQoL of patients with different clinical conditions undergoing PBM [10-13], there are no studies on the HRQoL of people with AGA. Physicians need to regard the importance of the psychological demands of people with AGA and the respective negative effects on their HRQoL and offer relevant treatment not only for hair loss but also for associated psychological distress [14].

We aimed to assess the HRQoL of men and women with AGA before and after PBM. We hypothesize that there is an improvement in HRQoL of individuals with AGA undergoing PBM.

## Materials and methods

This single-center prospective observational study was conducted in a city in the southeastern countryside of Brazil with approximately 130,000 inhabitants. The Research Ethics Committee of the Federal University of Uberlandia approved this study in 2019 at number 06298818.1.0000.5152 and informed that all study participants signed a written consent form.

We had a convenience sample of men and women with AGA from the local community. We included men with IIa, II, IIIa, III, IIIv, IVa, IV, Va, and V grades on the Norwood-Hamilton scale[15], and women with I-2, I-3, I-4, II-1, and II-2 grades of the Savin scale[16]. To participate in the study, men and women should be literate, aged between 18 and 60 years, have Fitzpatrick's skin phototypes from I to IV, be of any race/ethnicity, and do not have a history of scalp cancer, and initiation or use in the past six months of another treatment, such as minoxidil, finasteride (or any other 5-alpha-reductase inhibitor), medications with anti-androgenic properties (cyproterone acetate, spironolactone, ketoconazole, flutamide, bicalutamide), topical estrogen, progesterone, tamoxifen, anabolic steroids, medications that can cause hypertrichosis (cyclosporine, diazoxide, phenytoin, psoralens), oral corticosteroids (inhalants were allowed), lithium, phenothiazines, or serenoa repens (saw palmetto). Exclusion criteria included pregnancy, breastfeeding, and participants who attended less than 75% of PBM sessions.

The diagnosis of AGA and its classification was made by inspection of participants' hair and scalp. Trichoscopy was used in the diagnosis, which included hair shaft thickness heterogeneity of at least 20%, more than 10% of vellus hairs, a high number of follicular units with only one hair, yellow dots, peripilar sign, empty follicles, honeycomb pigment pattern, and prevalence of these changes in the frontal area compared to the occipital area [17].

Data collection occurred between June 2019 and August 2019. The recruitment period was the last two weeks of May 2019. At the beginning of the study, all participants answered a sociodemographic questionnaire in an interview; then, they individually answered the Brazilian version of Skindex-29 (self-application) [18]. To minimize the risk of identification, random codes were applied to each participant. The reliability and validity of the Skindex-29 questionnaire for Brazilian Portuguese were published by Paula et al. (2014) [18].

The Skindex-29 is a dermatological HRQoL instrument that comprehensively assesses the effects of skin diseases on HRQoL and identifies changes through time. The questionnaire covers three domains (emotions, symptoms, and psychosocial functioning). It comprises 29 items with a 5-point Likert scale with the following answers: never, rarely, sometimes, often, and all the time [19]. The final score is established either by the mean of the points obtained in the 29 items (total score) or by the mean of each domain (domain score).

The scores are standardized from 0 (no effect) to 100 (maximal effect), with higher scores indicating worse HRQoL [19]. We assessed all measurement properties from the consensus-based standards for the selection of health measurement instruments (COSMIN) checklist in this study.

After 24 PBM sessions - two 20-minute sessions per week, with 48 to 72 hours of interval between the sessions, the study participants individually answered the Skindex-29 again. For the PBM sessions, we used the iGrow Laser/LED helmet (Apira Science Inc., Boca Raton, USA), FDA 510 (k) approval, with 21 5-mW lasers (655 nm wavelength +/- 5 nm) and 30 LEDs (655 nm +/- 20 nm).

Missing data were managed as follows: a Skindex-29 score was not analyzed if 50% or more items in a domain/scale were missing[19]. For the missing items analyzed within each domain (emotions, psychosocial functioning, and symptoms), we used an imputation technique, and assigned the mean of the participant's responses to the remaining items for that domain, according to published protocols [19,20].

The sample size was calculated using a two-tailed test to demonstrate a medium effect size on major variables at 80% of statistical power, 5% of maximum type I error, and an estimated loss of 20% (G* Power 3.1.9.2 Franz Faul, Universität Kiel, Germany), considering a different prevalence for men and women that increased according to age. The initial prevalence among men was 18.5% in the population aged 18-19 years, and 60% for those aged 60 years; among women, the initial prevalence was 10% for those aged 18-19 years, and 29.22% for participants aged 60 years. The mean prevalence in the sample was 28.03% considering men and women aged between 18 and 60 years[21], resulting in a total of 85 recruited participants (42 men and 43 women).

Descriptive statistics were used to characterize the study sample. The Skindex-29 scores were compared using Student's t-test for paired samples according to the total scale and its domains (emotions, symptoms, and psychosocial functioning) before and after PBM. Clinical and sociodemographic characteristics were analyzed separately by gender. Skindex-29 score comparisons were analyzed separately by gender and with all participants together. We set a p-value of <0.05 for all statistical analyses.

Additionally, we calculated the magnitude of the statistically significant differences (effect size) from the determination of Cohen's d (ratio between the difference of means and the mean standard deviation for comparisons between two dependent groups). We interpret the following values as small, medium, or large for Cohen's d as 0.2; 0.5, and 0.8, respectively[22]. Medium effect sizes are generally considered to be clinically important[23]. We calculated Cohen's d for comparisons between T0 (before PBM) and T1 (after PBM). Data were analyzed using IBM SPSS Statistics for Windows, Version 25 (Released 2017; IBM Corp., Armonk, New York, United States). The internal consistency of Skindex-29 total scores and its domains was measured with Cronbach’s alpha coefficient for men and women.

## Results

**Figure 1:**
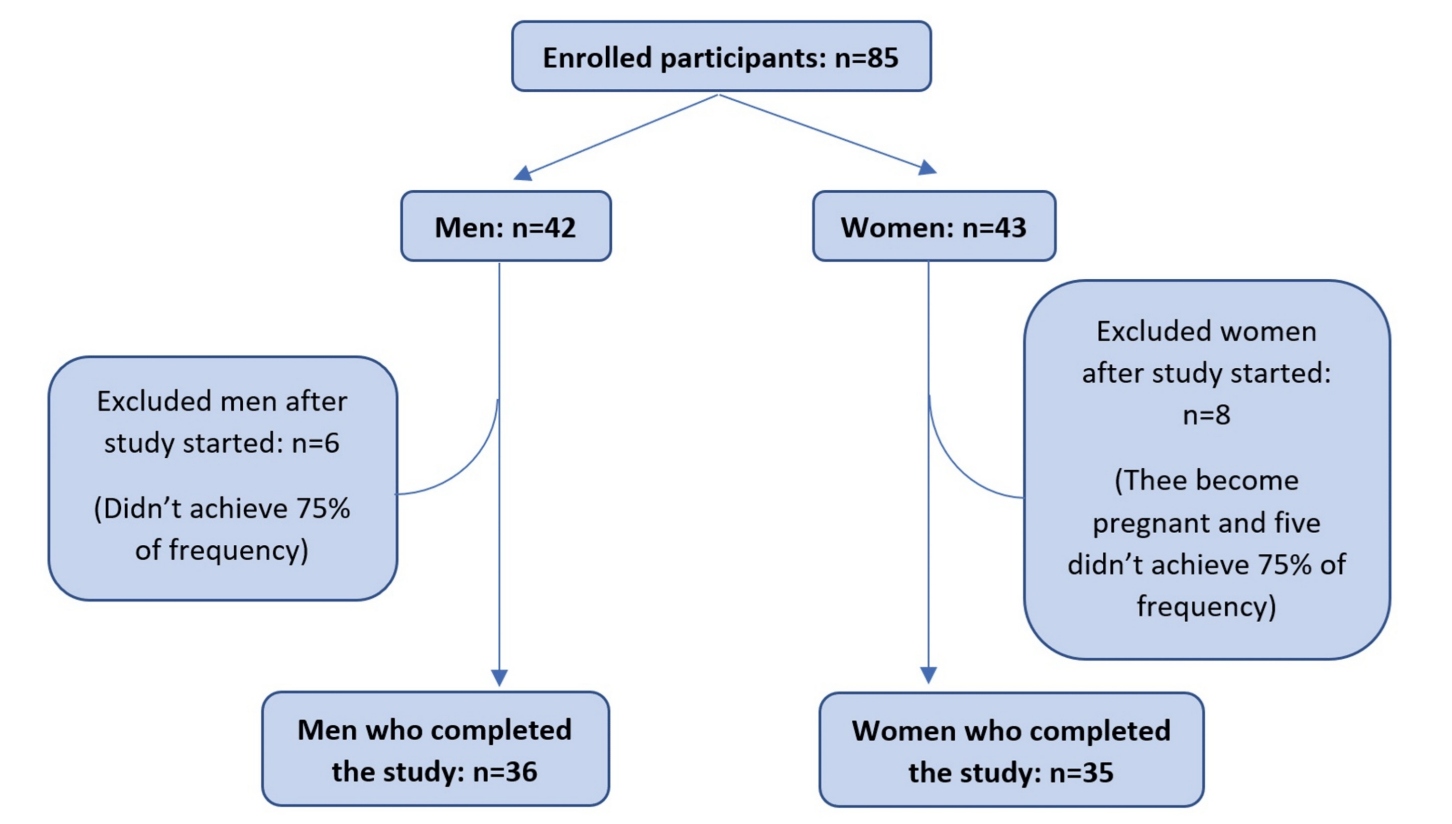
Fluxogram of study participants Source: Author’s original

Out of 85 enrolled individuals who met the inclusion criteria (42 men and 43 women), 71 completed the study (36 men and 35 women) (Figure 1).

Men had a mean age of 37.3 years (SD=9) and women 44.8 years (SD=11.8), a mean age of onset of 26.2 years (SD=7.4) for men, 35.3 years (SD=13) for women, and a mean disease duration of 11 years (SD=6.1) in men and 9.8 years (SD=8) in women. Most of the men were white (n=30; 83.3%) as well as women (n=22; 62.9%).

Men presented the Norwood-Hamilton grade IV (n=12; 33.3%) and IIIv (n=9; 25%), and women showed Savin degree I-2 (n=10; 28.6%) and II-2 (n=10; 28.6%). Most of the men corresponded to the Fitzpatrick Skin Phototype III (n=17; 47.2%) and II (n=10; 27.8%) and women III (n=13; 37.1%) and II (n=12; 34.3%) (Table 1).

**Table 1:** Clinical and sociodemographic characteristics of participants according to gender ^†^: mean years; SD: standard deviation; ^‡^: does not apply Men are graded according to the Norwood-Hamilton scale and women according to the Savin scale. Source: Author’s original

		Men (n=36)	Women (n=35)	
Age^†^ (SD)		37.3 (9)	44.8 (11.8)	
Age of disease onset^†^ (SD)		26.2 (7.4)	35.3 (13)	
Disease duration^†^ (SD)		11 (6.1)	9.8 (8)	
Grade: n (%)	IIa	2 (5.6)	I-2	10 (28.6)
	II	1 (2.8)	I-3	8 (22.9)
	IIIa	2 (5.6)	I-4	2 (5.7)
	III	4 (11.1)	II-1	5 (14.3)
	IIIv	9 (25.0)	II-2	10 (28.6)
	IVa	2 (5.6)	^‡^	^‡^
	IV	12 (33.3)	^‡^	^‡^
	Va	1 (2.8)	^‡^	^‡^
	V	3 (8.3)	^‡^	^‡^
Skin color	White	30 (83.)	22 (62.9)	
	Yellow	1 (8)	1 (2.9)	
	Brown	4 (11.1)	10 (28.6)	
	Black	1 (2.8)	2 (5.7)	
Fitzpatrick’s skin phototype	1	3 (8.3)	3 (8.6)	
	2	10 (27.8)	12 (34.3)	
	3	17 (47.2)	13 (37.1)	
	4	6 (16.7)	7 (20.0)	
Marital status	Married	25 (69.4)	23 (65.7)	
	Single	11 (30.6)	5 (14.3)	
	Divorced	0	2 (5.7)	
	Widower	0	5 (14.3)	

Excluded men had a mean age of 41.2 years (SD=14.6) and women 37.4 years (SD=10.8). Men presented Norwood-Hamilton grade II (n=1; 16.6%), IIIv (n=2; 33.3%), IVa (n=1; 16.6%), IV (n=1; 16.6%) and Va (n=1; 16.6%). Women presented Savin degree I-2 (n=3; 37.5%), I-3 (n=2; 25%), I-4 (n=1; 12.5%) and II-1 (n=2; 25%).

Missing data were found in 0.43% of items among men and in 0.74% of items among women. No participant was excluded for leaving more than 50% of items of each Skindex-29 domain unanswered.

Women had a large reduction in Skindex-29 total score after PBM (p<0.01; Cohen's d=0.82). When analyzing the Skindex-29 domains, women had large reductions in the emotions domain after PBM (p<0.01; Cohen's d=0.89), but also presented lower scores in the psychosocial functioning (p<0.01; Cohen's d=0.60) and symptoms (p=0.03; Cohen's d=0.38) domains (Table 2).

**Table 2:** Women’s Skindex-29 scores before and after photobiomodulation (PBM) (n=35) T0: before PBM; T1: after PBM; SD: standard deviation; t: t-value; df: degrees of freedom; p^*^: test of paired samples; CI: confidence interval; α: Cronbach’s alpha coefficient Source: Author’s original

SKINDEX-29 domains	T0 mean (SD)	T1 mean (SD)	t	df	p^*^	95% CI	α	Cohen’s d
Emotions	40.50 (26.01)	19.50 (22.99)	5.266	34	<0.01	[12.89, 29.10]	0.943	0.89
Symptoms	23.97 (18.13)	18.36 (12.96)	2.242	34	0.03	[0.52, 10.69]	0.861	0.38
Psychosocial functioning	16.90 (21.09)	7.85 (16.96)	3.449	34	<0.01	[3.88, 14.21]	0.952	0.60
SKINDEX-29 total	26.74 (19.37)	14.40 (16.67)	4.847	34	<0.01	7.16, 17.51]	0.961	0.92

Men presented a moderate reduction in Skindex-29 total score after PBM (p<0.01; Cohen's d=0.68). When analyzing the Skindex-29 domain scales, men had largely lower scores in the emotion domain after PBM (p<0.01; Cohen's d=0.82) and small reductions in the psychosocial functioning (p<0.01; Cohen's d=0.47) score (Table 3).

**Table 3:** Men’s Skindex-29 scores before and after photobiomodulation (PBM) (n=36) T0: before PBM; T1: after PBM; SD: standard deviation; t: t-value; df: degrees of freedom; p^*^: test of paired samples; CI: confidence interval; α: Cronbach’s alpha coefficient; ⁗: not calculated due to p>0.05 PBM: photobiomodulation Source: Author’s original

SKINDEX-29 domains	T0 mean (SD)	T1 mean (SD)	t	df	p^*^	95% CI	α	Cohen’s d
Emotions	26.94 (20.94)	7.63 (11.17)	4.905	35	<0.01	[11.31, 27.29]	0.922	0.82
Symptoms	18.35 (17.20)	12.00 (10.06)	1.942	35	0.06	[-0.289, 12.98]	0.844	⁗
Psychosocial functioning	11.28 (14.52)	3.41 (7.48)	2.831	35	<0.01	[2.22, 13.51]	0.910	0.47
SKINDEX-29 total	18.38 (14.32)	6.94 (7.88)	4.067	35	<0.01	[5.73, 17.15]	0.937	0.68

## Discussion

In this study, we observed important gender differences in HRQoL in the AGA population. Women had larger impacts in all Skindex-29 domains and an expressive improvement in the emotional domain of HRQoL. Gender differences in HRQoL related to AGA are controversial in the literature[24], probably due to the different roles that hair plays in cultures. The emotional importance of hair for women begins in their childhood. Hair is related to women's identity and plays an important role in physical attractiveness, beauty, health, fertility, and youth in many Western and Eastern cultures. Cultural factors include shared beliefs and behaviors as culture shapes a pattern of learned beliefs, communication styles, interactions, societal roles, values, and practices. Psychological factors consider emotional well-being, psychological states such as stress, anxiety, depression, and social environment. These factors associated with hair loss in women could significantly affect HRQoL [25]. Another possible explanation for the gender differences found in our study is the higher prevalence of AGA in men [26].

Male AGA is usually more accepted by both society and men themselves when compared to women [27]. This is probably why women in our study showed moderate improvement in the symptoms domain whereas men did not. Some limitations also need consideration. An observational design may not provide results with high levels of evidence like experimental studies do. Further randomized controlled trials (RCTs) should be pursued following our findings. Although our results come from a single-center sample, the generalizability is founded on the number and heterogeneity of the sample.

In the psychosocial functioning domain, men and women showed improvement in HRQoL compared to before and after PBM. Reductions in the AGA effect on self-esteem and psychological adjustment are more evident among women than men[14]. The psychological impact of AGA is more severe on women than on men. More important psychosocial changes are expected in women with AGA when compared to men due to the lower prevalence of the disease in women, as well as the cultural patterns of beauty concerning women's hair[27].

Given the psychological and symbolic importance of hair, hair loss can have a potentially adverse impact on the HRQoL of individuals affected by AGA. Unfortunately, this impact is often trivialized or even ignored by those not affected by this condition. Some studies have verified the psychological difficulties experienced by men with AGA. Men with visible hair loss are generally seen by others as significantly older, physically or socially less attractive, weaker, and less powerful than their peers [28].

Our study has several strengths. It assesses HRQoL in the treatment of a condition that strongly affects HRQoL, considering that AGA has no cure. To the best of our knowledge, there are no other studies assessing HRQoL from the perspective of individuals with AGA undergoing PBM. The self-controlled method used has the advantage of managing all fixed confounders, which do not vary over time [29].

Some limitations also need consideration. Although there are divergences in the literature regarding the levels of evidence of an observational study[30]. Further observational and RCTs should be pursued following our findings. Though our results come from a single-center sample, the generalizability is founded on the number and heterogeneity of the sample. The selection bias was minimized by considering the careful patient selection method and the sample size suggested by the software G*Power could be a factor of selection bias, considering the high prevalence of AGA in the population. The reporting bias was also minimized with an effort to transparent reporting and taking into consideration the impact of overall evidence. The analysis does not include multivariable models that could account for potential confounding variables, such as age and duration of AGA.

## Conclusions

In this study, we demonstrated that the use of PBM in AGA is associated with an improvement in the HRQoL of men and women. This enhancement is even higher concerning emotions, the major domain affected in the AGA population. Women had larger impacts in all Skindex-29 domains after undergoing PBM. This study helps bridge a gap in knowledge concerning the use of PBM in AGA and its effects on patients' health-related HRQoL. Patients' perceptions of HRQoL, in addition to dermatologists', clinicians', or external observers' opinions, should be taken into consideration in any treatment related to AGA.

Future research related to PBM in AGA should include long-term follow-up studies, considering the possibility of providing valuable data on the durability of HRQoL improvements, and RCTs to evaluate the amelioration of HRQoL associated with PBM compared to other treatments or placebo.

## References

[1] Zhuang XS, Zheng YY, Xu JJ, Fan WX (2013). Quality of life in women with female pattern hair loss and the impact of topical minoxidil treatment on quality of life in these patients. Exp Ther Med.

[2] Alfonso M, Richter-Appelt H, Tosti A, Viera MS, García M (2005). The psychosocial impact of hair loss among men: a multinational European study. Curr Med Res Opin.

[3] Cartwright T, Endean N, Porter A (2009). Illness perceptions, coping and quality of life in patients with alopecia. Br J Dermatol.

[4] Reid EE, Haley AC, Borovicka JH, Rademaker A, West DP, Colavincenzo M, Wickless H (2012). Clinical severity does not reliably predict quality of life in women with alopecia areata, telogen effluvium, or androgenic alopecia. J Am Acad Dermatol.

[5] Van Der Donk J, Hunfeld JA, Passchier J (1994). Quality of life and maladjustment associated with hair loss in women with alopecia androgenetica. Soc Sci Med.

[6] Girman CJ, Rhodes T, Lilly FR, Guo SS, Siervogel RM, Patrick DL, Chumlea WC (1998). Effects of self-perceived hair loss in a community sample of men. Dermatology.

[7] Gupta AK, Foley KA (2017). A critical assessment of the evidence for low-level laser therapy in the treatment of hair loss. Dermatol Surg.

[8] Avci P, Gupta GK, Clark J, Wikonkal N, Hamblin MR (2014). Low-level laser (light) therapy (LLLT) for treatment of hair loss. Lasers Surg Med.

[9] Yim E, Nole KL, Tosti A (2014). 5α-Reductase inhibitors in androgenetic alopecia. Curr Opin Endocrinol Diabetes Obes.

[10] Gur A, Sarac AJ, Cevik R, Altindag O, Sarac S (2004). Efficacy of 904 nm gallium arsenide low level laser therapy in the management of chronic myofascial pain in the neck: a double-blind and randomize-controlled trial. Lasers Surg Med.

[11] Campos L, Simões A, Sá PH, Eduardo Cde P (2009). Improvement in quality of life of an oncological patient by laser phototherapy. Photomed Laser Surg.

[12] Gautam AP, Fernandes DJ, Vidyasagar MS, Maiya AG, Nigudgi S (2013). Effect of low-level laser therapy on patient reported measures of oral mucositis and quality of life in head and neck cancer patients receiving chemoradiotherapy--a randomized controlled trial. Support Care Cancer.

[13] Sezer U, Eltas A, Ustün K, Senyurt SZ, Erciyas K, Aras MH (2012). Effects of low-level laser therapy as an adjunct to standard therapy in acute pericoronitis, and its impact on oral health-related quality of life. Photomed Laser Surg.

[14] Cash TF (1999). The psychosocial consequences of androgenetic alopecia: a review of the research literature. Br J Dermatol.

[15] Norwood OT (1975). Male pattern baldness: classification and incidence. South Med J.

[16] Savin RC (1992). A method for visually describing and quantitating hair loss in male pattern baldness. J Invest Dermatol.

[17] Rakowska A, Slowinska M, Kowalska-Oledzka E, Olszewska M, Rudnicka L (2009). Dermoscopy in female androgenic alopecia: method standardization and diagnostic criteria. Int J Trichology.

[18] Paula HR, Haddad A, Weiss MA, Dini GM, Ferreira LM (2014). Translation, cultural adaptation, and validation of the American Skindex-29 quality of life index. An Bras Dermatol.

[19] Chren MM, Lasek RJ, Flocke SA, Zyzanski SJ (1997). Improved discriminative and evaluative capability of a refined version of Skindex, a quality-of-life instrument for patients with skin diseases. Arch Dermatol.

[20] Ware JE (1993). Measuring patients' views: the optimum outcome measure. BMJ.

[21] Gan DC, Sinclair RD (2005). Prevalence of male and female pattern hair loss in Maryborough. J Investig Dermatol Symp Proc.

[22] Cohen J (1988). Statistical Power Analysis for the Behavioral Sciences.

[23] Sloan JA, Dueck A (2004). Issues for statisticians in conducting analyses and translating results for quality of life end points in clinical trials. J Biopharm Stat.

[24] Jun M, Keum DI, Lee S, Kim BJ, Lee WS (2018). Quality of life with alopecia areata versus androgenetic alopecia assessed using hair specific skindex-29. Ann Dermatol.

[25] Hirschman EC, Brunswick N (2002). Hair as attribute, hair as symbol, hair as self. Gender and Consumer Behavior.

[26] Norwood OT (2001). Incidence of female androgenetic alopecia (female pattern alopecia). Dermatol Surg.

[27] Cash TF, Price VH, Savin RC (1993). Psychological effects of androgenetic alopecia on women: comparisons with balding men and with female control subjects. J Am Acad Dermatol.

[28] Passchier J (1998). Quality of life issues in male pattern hair loss. Dermatology.

[29] Whitaker HJ, Farrington CP, Spiessens B, Musonda P (2006). Tutorial in biostatistics: the self-controlled case series method. Stat Med.

[30] Concato J, Shah N, Horwitz RI (2000). Randomized, controlled trials, observational studies, and the hierarchy of research designs. N Engl J Med.

